# Inhibition of human tumour prostate PC-3 cell growth by cannabinoids R(+)-Methanandamide and JWH-015: Involvement of CB_2_

**DOI:** 10.1038/sj.bjc.6605248

**Published:** 2009-08-18

**Authors:** N Olea-Herrero, D Vara, S Malagarie-Cazenave, I Díaz-Laviada

**Affiliations:** 1 Department of Biochemistry and Molecular Biology, School of Medicine, University of Alcalá, Alcalá de Henares, 28871 Madrid, Spain

**Keywords:** cannabinoids, CB_2_ receptor, ceramide, PC-3 cells, prostate cancer

## Abstract

**Background:**

We have previously shown that cannabinoids induce growth inhibition and apoptosis in prostate cancer PC-3 cells, which express high levels of cannabinoid receptor types 1 and 2 (CB_1_ and CB_2_). In this study, we investigated the role of CB_2_ receptor in the anti-proliferative action of cannabinoids and the signal transduction triggered by receptor ligation.

**Methods:**

The human prostate cancer cell lines, namely PC-3, DU-145 and LNCaP, were used for this study. Cell proliferation was measured using MTT proliferation assay, [^3^H]-thymidine incorporation assay and cell-cycle study by flow cytometry. Ceramide quantification was performed using the DAG kinase method. The CB_2_ receptor was silenced with specific small interfering RNA, and was blocked pharmacologically with SR 144528. *In vivo* studies were conducted by the induction of prostate xenograft tumours in nude mice.

**Results:**

We found that the anandamide analogue, R(+)-Methanandamide (MET), as well as JWH-015, a synthetic CB_2_ agonist, exerted anti-proliferative effects in PC-3 cells. R(+)-Methanandamide- and JWH-015-induced cell death was rescued by treatment with the CB_2_ receptor antagonist, SR 144528. Downregulation of CB_2_ expression reversed the effects of JWH-015, confirming the involvement of CB_2_ in the pro-apoptotic effect of cannabinoids. Further analysing the mechanism of JWH-015-induced cell growth inhibition, we found that JWH-015 triggered a *de novo* synthesis of ceramide, which was involved in cannabinoid-induced cell death, insofar as blocking ceramide synthesis with Fumonisin B1 reduced cell death. Signalling pathways activated by JWH-015 included JNK (c-Jun N-terminal kinase) activation and Akt inhibition. *In vivo* treatment with JWH-015 caused a significant reduction in tumour growth in mice.

**Conclusions:**

This study defines the involvement of CB_2_-mediated signalling in the *in vivo* and *in vitro* growth inhibition of prostate cancer cells and suggests that CB_2_ agonists have potential therapeutic interest and deserve to be explored in the management of prostate cancer.

Prostate cancer has become the most common cancer diagnosed in men and is one of the major life-threatening diseases in Western countries (Ukraintseva *et al*, 2008). Despite recent advances in its diagnosis and treatment, current therapies are unable to completely eliminate the androgen-independent prostate cancer cells that remain after androgen ablation therapy (Bahnson, 2007). Thus, understanding the mechanisms involved in the control of tumour growth and the development of chemopreventive agents are major goals of basic research in oncology.

Cannabinoids, the active components of *Cannabis sativa* and their derivatives, exert a wide spectrum of modulatory actions and pharmacological activities in the brain as well as in the periphery, and therefore, the therapeutic potential of cannabinoids has gained much attention during the past few years (Kogan and Mechoulam, 2007). One of the most exciting areas of current research in the therapeutic potential of cannabinoids is cancer. Recent evidence suggests that cannabinoids are powerful regulators of cell growth and differentiation. They have been shown to exert anti-tumoural effects by decreasing viability, proliferation, adhesion and migration on various cancer cells, thereby suggesting the potential use of cannabinoids in the treatment of gliomas, prostate and breast cancers and malignancies of immune origin (Bifulco *et al*, 2006; Flygare and Sander, 2008; Sarfaraz *et al*, 2008; Pisanti *et al*, 2009). The mechanisms of the anti-tumoural action of cannabinoids include inhibition of tumour cell proliferation, induction of cell death, inhibition of cell migration and metastasis, anti-angiogenic effects and modulation of immune response (Guzman, 2003; Flygare and Sander, 2008). Various cannabinoids, especially anandamide and THC, promote apoptosis of astrocytoma, glioma, neuroblastoma and pheochromocytoma cells in culture by a pathway involving cannabinoid receptors (Guzman, 2003; Goncharov *et al*, 2005; Velasco *et al*, 2007) and by an activation of the reticulum stress pathway (Carracedo *et al*, 2006b; Salazar *et al*, 2009a). Moreover, synthetic as well as naturally occurring cannabinoids inhibit the growth of endocrine-related cancer cells, such as thyroid, breast and prostate cancer cells (revised in Lopez-Rodriguez *et al* (2005) and Bifulco *et al* (2008)). The antitumour effect of cannabinoids has been demonstrated both *in vitro* and *in vivo*, affecting multiple signalling pathways and biological processes that have been implicated in the development of the malignant phenotype (Bifulco *et al*, 2008). Cannabinoids may target tumour cells by binding to cannabinoid receptors or by a receptor-independent mechanism. Two cannabinoid receptors have been identified by molecular cloning to date: the CB_1_ receptor, which is highly expressed in the brain and also present in peripheral tissues, and the CB_2_ receptor, previously believed to be expressed primarily in immune and haematopoietic cells, although more recent studies have also identified CB_2_ receptors in the brain and in endothelial cells of various origins (Pertwee, 2006; Mackie, 2008). The CB_2_ receptor is unrelated to cannabinoid psychoactivity, and therefore targeting this receptor is one of the major challenges in cannabinoid therapeutic research (Ashton *et al*, 2008). After an interaction with cannabinoid receptors, cannabinoids trigger several signalling pathways depending on cell type, which may be involved in their anti-proliferative effects such as p38 MAPK and c-Jun N-terminal kinase (JNK) activation, increased synthesis of the pro-apoptotic sphingolipid ceramide and several downstream stress-related genes expressed in the endoplasmic reticulum (Diaz-Laviada and Ruiz-Llorente, 2005; Demuth and Molleman, 2006). Ceramide, the central molecule of sphingolipid metabolism, generally mediates anti-proliferative responses, such as inhibition of cell growth, induction of apoptosis and/or modulation of senescence (Saddoughi *et al*, 2008). Several therapeutic agents that induce ceramide-dependent apoptosis in cancerous cells currently exist, and a number of enzymes involved in ceramide metabolism are beginning to be recognised as potential targets for cancer therapy (Savtchouk *et al*, 2007; Carpinteiro *et al*, 2008). Recent research has shown that cannabinoids induce ceramide accumulation in cancer cells, which is related to the pro-apoptotic effect of cannabinoids in cancer cells (Guzman *et al*, 2001; Velasco *et al*, 2005).

Prostate cancer cells express both CB_1_ and CB_2_ receptors (Ruiz-Llorente *et al*, 2003; Sanchez *et al*, 2003), the expression of which is significantly higher than that in normal prostate epithelial cells (Sarfaraz *et al*, 2005). The impact of cannabinoids on prostate cancer cell viability is variable and depends on the dose used. Low doses below the micromolar range induce androgen receptor expression in the prostate cell line, LNCaP (Sanchez *et al*, 2003), whereas doses in the range of micromolar or higher induce apoptosis (Ruiz *et al*, 1999; Mimeault *et al*, 2003) or cell-cycle arrest (Sarfaraz *et al*, 2006). The involvement of cannabinoid receptors in such effects is uncertain as CB_1_ antagonists blocked cytotoxic effects at short incubation times (Mimeault *et al*, 2003; Sarfaraz *et al*, 2005), although there was a lack of effect at a longer duration (Ruiz *et al*, 1999; Mimeault *et al*, 2003).

In this study, we analysed the role of CB_2_ receptor in the androgen-resistant prostate cell line, PC-3, which represents the androgen-refractory phase of advanced prostate cancer. We used the anandamide analogue, R(+)Methanandamide (MET), for comparison with previous results, and a potent and selective CB_2_ receptor agonist, JWH-015 (JWH), as well as CB_2_ antagonists and RNA silencing to show the role of CB_2_ in PC-3 cells.

## Materials and methods

### Reagents

R(+)-Metanandamide, JWH-015 [(2-methyl-1-propyl-1*H*-indol-3-yl)-1-naphtalenylmethanone] and Fumonisin B1 were purchased from Sigma (St Louis, MO, USA). SR 144528 (SR2) was kindly provided by Sanofi Recherche (Montpelier, France). Antibody anti-CB_2_ receptor was obtained from Affinity BioReagents (Golden, CO, USA). We purchased anti-tubulin, anti-phospho Akt at Ser473, anti-phospho p38, anti-total p38, anti-phospho JNK, anti-total JNK, anti-phosphor eIF2*α*, anti-caspase 8 and anti-caspase 9 and anti-citochrome *c* antibodies from Cell Signaling Technology (St Louis, MO, USA). The inhibitor D609 and the enzyme diacylglycerol kinase were supplied by Calbiochem (La Jolla, CA, USA).

### Cell cultures

Human prostate epithelial PC-3, DU-145 and LNCaP cells were purchased from American Type Culture Collection (Rockville, MD, USA) and were cultivated in RPMI-1640 medium supplemented with 10% fetal calf serum (FCS), 100 U ml^−1^ penicillin G, 100 *μ*g ml^−1^ streptomycin and 0.25 *μ*g ml^−1^ amphotericin B (Invitrogen, Paisley, UK). Low cell passages (between 10 and 20) were used for this study. Cells were seeded sub-confluently and, 1 day before the experiment, the serum was removed to work with quiescent cells.

### Cell viability assays

Cells were set up 2 × 10^4^ cells per well of a 24-well plate and were cultured in the RPMI 1640 medium supplemented with 10% FCS. After treatments according to figure legends, cell viability was assayed by MTT as previously described (Sanchez *et al*, 2003).

### Flow cytometry

Flow cytometry was used to detect apoptotic cells and the distribution of cell cycle. After being cultivated with medium alone or medium containing the indicated stimuli, 10^5^ cells in a 35-mm culture dish were harvested in 0.1% Nonidet P-40 and 0.5 mg ml^−1^ Rnase for 30 min and stained with 0.05 mg ml^−1^ Iodure Propidium (IP) to indicate the relative DNA content. The sub-G1 peak (DNA content <2 N) and cell-cycle distribution were measured using FACScan flow cytometer (Becton Dickinson, Franklin Lakes, NJ, USA). To analyse apoptosis by Annexin V staining, the cells were washed twice with PBS and incubated in 0.5 ml of binding buffer (10 mM HEPES pH 7.4, 150 mM NaCl, 2.5 mM CaCl_2_, 1 mM MgCl_2_ and 4% BSA), with 4 *μ*g ml^−1^ Annexin V-FITC for 15 min. The cells were then washed in PBS and re-suspended in the binding buffer with 0.6 *μ*g ml^−1^ IP (Calbiochem). In all, 20 000 cells of each sample were analysed by flow cytometry in a FACScan (Beckton Dickinson).

### Measurement of [^3^H]-thymidine incorporation into DNA

Cells were treated with different concentrations of MET or JWH-015 according to the experiment. DNA synthesis was determined by pulsing the cells with [^3^H]-thymidine (1 *μ*Ci per well) during the last 16 h of the culture period as previously described (Sanchez *et al*, 2003).

### siRNA tranfections

Cells were transfected in 1 ml OPTIMEN containing 4 *μ*g lipofectamine 2000 (Invitrogen Co., Carlsbad, CA, USA), with 100 nM human cannabinoid receptor-2-specific small interfering RNA (siRNA) duplexes (5′-GGCCUCUUCCCAAUUUAAAtt-3′, Applied Biosystems, Foster City, CA, USA) or control scrambled RNA for 12 h according to the manufacturer's protocols (Ambion, Applied Biosystems). At 24 h after transfection, the medium was removed and replaced with RPMI 1640 with 10% FCS medium. At dedicated time points after transfection, the cells were used for flow cytometry assays or western blot.

### Measurement of ceramide levels

Ceramide quantification in cell lipid extracts was performed according to the method described by Perry *et al* for ceramide quantification in cultured cells (Perry *et al*, 2000). Briefly, cell pellets were suspended in 0.6 ml distilled water, and disrupted at 4 °C by sonication. Lipids were extracted with chloroform/methanol, and ceramide content was determined by phosphorylation using *Escherichia coli* diacylglycerol kinase and [^32^P]*γ*-ATP (6000 Ci mmol^−1^; Perkin-Elmer, Barcelona, Spain). Products of this reaction were purified by TLC using chloroform–acetone–methanol–acetic acid–water (50 : 20 : 15 : 10 : 5, by volume) as the developing solvent. Products of this reaction were quantified and expressed as a percentage of the value observed earlier (Sanchez *et al*, 2007).

### Western blot analysis

Cultured cells were lysed into a lysis buffer (50 mM Tris-HCl, pH 7.4, 5 mM EDTA, 1 mM EGTA, 10 mM 2-mercaptoethanol) containing 5 *μ*g ml^−1^ leupeptin, 5 *μ*g ml^−1^ aprotinin and 1 mM phenylmethylsulfonyl fluoride, and were disrupted by sonication. Protein concentration was determined using the Bio-Rad protein assay (Bio-Rad Laboratories, Hercules, CA, USA). Western blotting was carried out as previously described (Sanchez *et al*, 2006).

### *In vivo* anti-tumour activity

All animal studies were conducted in accordance with the Spanish institutional regulation for the housing, care and use of experimental animals, have been carried out with ethical committee approval and met the European Community directives regulating animal research. Recommendations made by the UKCCCR have been adhered to carefully. Athymic nude (nu/nu) 6-week-old male mice were purchased from Harlan Iberica (Barcelona, Spain) and were housed in a laminar airflow cabinet under pathogen-free conditions on a 12-h light–dark schedule. Mice were injected subcutaneously (s.c.) in the right flank with 2 × 10^6^ PC-3 cells in 0.2 ml of complete culture medium. Two weeks after transplantation, tumours had grown to an average volume of 70 mm^3^. Mice were then divided into three experimental groups of eight animals each, which received the following treatments as s.c. injections: group A, saline (control); group B, 0.15 mg kg^−1^ body weight (b.w.) JWH-015; group C, 0.15 mg kg^−1^ b.w. JWH-015 plus 0.15 mg kg^−1^ b.w SR2. The injection was repeated every day and treatment was continued for 14 days. Tumour volumes were monitored every day using calliper measurements and were calculated using the following formula: (4*π*/3) × (*w*/2)^2^ × (*l*/2), where *w*=width and *l*=length. The b.w. of the animals was recorded daily.

### Statistical analysis

Data are presented as mean±s.e. of the number of experiments indicated. Statistical comparisons among groups were made with Student's *t*-test, and the difference was considered to be statistically significant when the *P*-value was <0.05.

## Results

### The cannabinoids, MET and JWH-015, inhibited cell growth of prostate cancer cells

**Figure 1:**
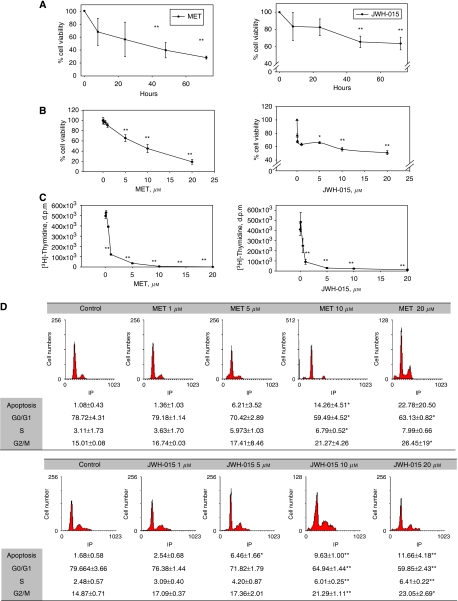
The anti-proliferative effect of the cannabinoids, R(+)-Methanandamide and JWH-015, on prostate PC-3 cells. (**A**) Time course of cannabinoid effect on prostate PC-3 cells viability. PC-3 cells were incubated with 10 *μ*M MET or 10 *μ*M JWH-015 for different times and cell viability was assayed by MTT. (**B**) Cells were incubated in the presence of increasing concentrations of MET or JWH-015 for 48 h and cell viability was assayed by MTT. (**C**) Cells were incubated in the presence of increasing concentrations of MET or JWH-015 for 48 h and cell proliferation was measured by [^3^H]-thymidine incorporation. (**D**) Cells were incubated in the presence of increasing concentrations of MET or JWH-015 for 48 h and cell cycle was assayed by flow cytometry. Data are the means±s.e. of three different experiments, each performed in triplicate. ^*^P<0.05 and ^**^P<0.01 using Student's *t*-test for the comparison between vehicle-treated and cannabinoid-treated cells.

**Figure 2:**
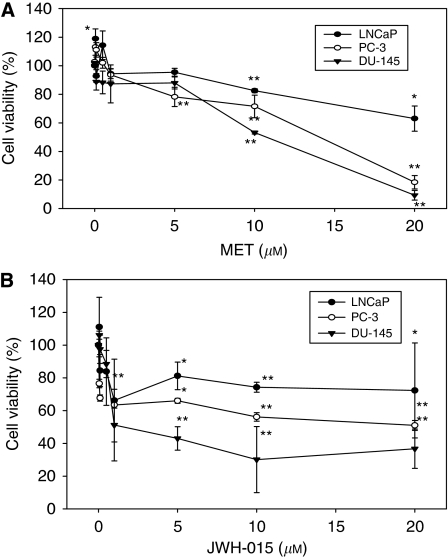
Anti-proliferative effect of cannabinoids on different prostate cancer cell lines. (**A**) Prostate cancer LNCaP, PC-3 or DU-145 cells were incubated with different doses of MET for 48 h and cell viability was assayed by MTT. (**B**) Prostate cancer LNCaP, PC-3 or DU-145 cells were incubated with different doses of JWH-015 for 48 h and cell viability was assayed by MTT. Data are means±s.e. of two different experiments, each performed in triplicate. ^*^P<0.05 and ^**^P<0.01 using Student's *t*-test for the comparison between vehicle-treated and cannabinoid-treated cells.

We first examined the anti-proliferative effects of the stable anandamide analogue, MET, and the synthetic CB_2_ ligand, JWH-015, on prostate PC-3 cells. The kinetics of MET and JWH-015 treatment showed that cannabinoid-induced cell death was evident from 12 h, although maximal effect was reached at 48–72 h (Figure 1A). Thus, we decided to follow all the studies at 48 h. Cells were incubated in the presence of increasing concentrations of MET or JWH-015 for 48 h, after which cell viability was evaluated by MTT assay, [^3^H]-thymidine incorporation assay or by flow cytometry. As shown in Figure 1B, both MET and JWH-015 caused a dose-dependent decrease in cell viability, which was significantly different from control from doses over 5 *μ*M. To assess the suppressive effects of R(+)-Methanandamide and JWH-015 on the proliferation of PC-3 cells, DNA synthesis was measured by [^3^H]-thymidine incorporation. Results shown in Figure 1C indicate that both cannabinoids inhibited the proliferation of PC-3 cells, which was totally blocked from doses over 5 *μ*M. The cell-cycle analysis demonstrated that cannabinoid treatment resulted in a small, although significant, accumulation of cells in the sub-G1 phase of the cell cycle (Figure 1D). These results suggest that the compounds used induced a small percentage of apoptosis and growth arrest in prostate cells. To investigate whether the anti-proliferative effect of cannabinoids on prostate cancer cells was generalised, we used the androgen-refractory prostate cancer DU-145 cells and the less tumourigenic androgen-dependent prostate LNCaP cells. Results shown in Figure 2 showed that both MET and JWH-015 inhibited the growth of the three cancer prostate lines studied, although the effect was less pronounced in the androgen-sensitive LNCaP cells. As shown in Figure 2A, low doses (sub-micromolar) of MET induced a slight increase in LNCaP cell viability, as previously reported by our group (Sanchez *et al*, 2003).

**Figure 3:**
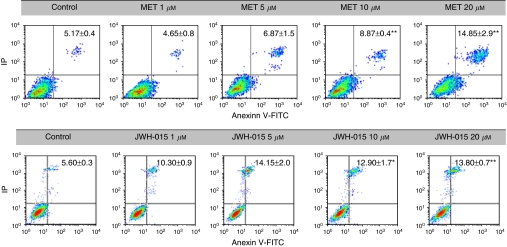
Evaluation of apoptosis by Annexin V-FITC/IP staining, followed by flow cytometry analysis. Representative plots of Annexin V-FITC/IP staining of PC-3 cells cultured in the presence of increasing concentrations of MET or JWH-015 are shown. Data showing the percentage of late apoptotic cells (upper right quadrant) are the mean±s.e. of three different experiments, each performed in duplicate. ^*^P<0.05 and ^**^P<0.01 using Student's *t*-test for the comparison between vehicle-treated and cannabinoid-treated cells.

To quantify the percentage of apoptotic cells after drug treatments, PC-3 cells were stained with Annexin V-FITC/IP. Results show that the rate of late apoptotic cells (Annexin V-FITC positive/IP positive, upper right quadrant) in MET- and JWH-015-treated cells was statistically increased compared with that in control cells (Figure 3). R(+)Methanandamide and JWH-15 treatments also induced cell necrosis, as inferred from IP-positive cells (upper left quadrant). Early apoptotic cells (Annexin V-FITC positive/IP negative, lower right quadrant) were <5% in all cases. These findings indicate that both MET and JWH-015 promoted a low, although significant, percentage of apoptosis in prostate cancer cells, but other processes such as mitotic catastrophe, cytotoxicity or necrosis could also collaborate in the observed growth inhibition.

### Involvement of CB_2_ in the anti-proliferative effect of cannabinoids

**Figure 4:**
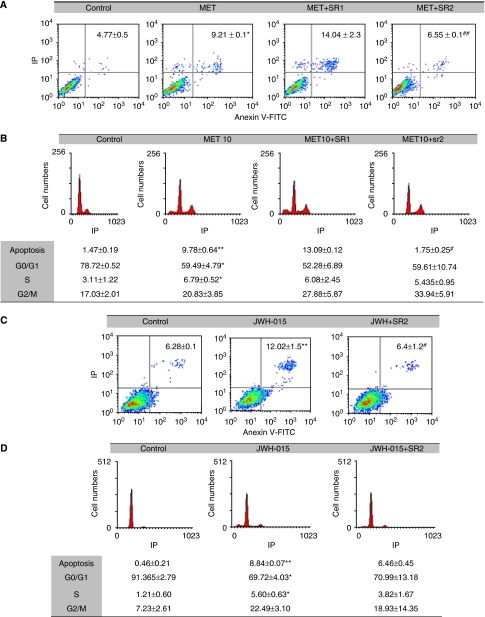
Inhibition of cannabinoid-induced cell death by the CB_2_ antagonist, SR 144528 (SR2). PC-3 cells were incubated with 10 *μ*M MET or 10 *μ*M JWH-015 for 48 h in the presence or absence of 0.5 *μ*M Rimonabant (SR1) or 2 *μ*M SR2. Apoptosis was assayed by Annexin V-FITC/IP staining (panels **A** and **C**) and cell cycle was measured by IP staining (panels **B** and **D**). Representative plots are shown in the figure and data are the mean±s.e. of three different experiments, each performed in duplicate. ^*^P<0.05 and ^**^P<0.01 using Student's *t*-test for the comparison between vehicle-treated and cannabinoid-treated cells, and ^#^*P*<0.05 and ^##^*P*<0.01 for the comparison between cannabinoid-treated and antagonist-treated cells.

As we have previously shown, prostate PC-3 cells express both CB_1_ and CB_2_ cannabinoid receptors (Sanchez *et al*, 2003). We then investigated the role of CB_1_ and CB_2_ in cannabinoid-induced prostate cell death. Pharmacological blockage of CB_1_ with its antagonist Rimonabant (SR1) did not reduce the effect of MET on cell cycle or apoptosis (Figure 4A). However, the CB_2_ antagonist, SR 144528 (SR2), reduced the number of apoptotic cells and the number of sub-G1 cells induced by MET treatment (Figure 4A). As MET is a weak ligand for CB_2_, we confirmed this result with the CB_2_-selective agonist JWH-015. The JWH-015-induced cell death effect was reverted by SR2, suggesting a role for CB_2_ in the apoptotic mechanisms of cannabinoids in PC-3 cells.

**Figure 5:**
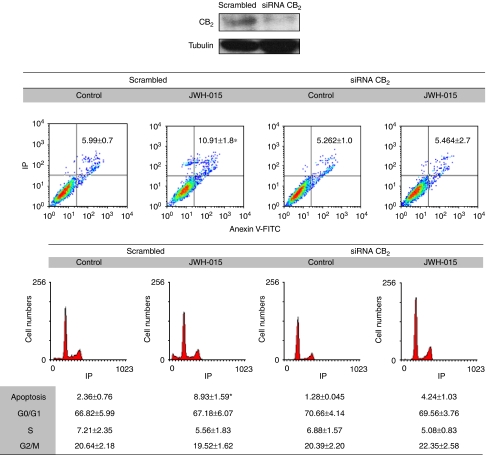
CB_2_ is involved in the anti-proliferative effect of JWH-015 in PC-3 cells. Cells were transfected with CB_2_-specific small interfering RNA (siRNA) or with control scrambled RNA for 48 h and then treated with 10 *μ*M JWH-015 for an additional 48 h. Cell apoptosis or cell cycle was assayed by flow cytometry. A representative plot is shown. Data are the mean±s.e. of two different experiments performed in duplicate. ^*^P<0.05 using Student's *t*-test for the comparison between control and JWH-015-treated cells. Upper panel, western blot for CB_2_ in control (scrambled) and CB_2_ siRNA-transfected cells.

To confirm the involvement of CB_2,_ we silenced its expression with siRNA. PC-3 cells were transfected with CB_2_-selective siRNA or control scrambled RNA for 48 h, after which the expression of CB_2_ was notably reduced as it was corroborated by western blotting (Figure 5). Under these conditions, apoptosis induced by 10 *μ*M JWH-015 was almost totally blocked in cells transfected with CB_2_ siRNA when compared with scrambled siRNA-transfected cells (Figure 5). These results confirm the involvement of CB_2_ receptor in the pro-apoptotic effect of cannabinoids in prostate cells. Therefore, we conducted the rest of the experiments with JWH-015, which is a potent and selective ligand for CB_2_ and which exhibits more efficacy than MET for CB_2_ activation.

### Ceramide synthesis mediates the anti-proliferative effect induced by CB_2_ activation

**Figure 6:**
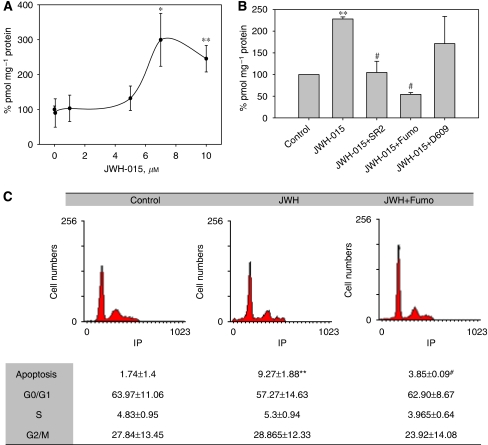
Involvement of ceramide synthesis in JWH-015-induced cell growth inhibition. (**A**) PC-3 cells were incubated in the presence of increasing concentrations of JWH-015 for 48 h and intracellular ceramide was measured by the DAG kinase method as indicated in the Methods section. (**B**) PC-3 cells were incubated with 10 *μ*M JWH-015in the presence or absence of 2 *μ*M SR2, 50 *μ*M Fumonisin B1 (Fumo) or 5 *μ*M D609 for 48 h and intracellular ceramide was assayed as above. (**C**) Cell cycle of PC-3 cells incubated with 10 *μ*M JWH-015±50 *μ*M Fumo for 48 h. Data are the mean±s.e. of three different experiments performed in duplicate. ^*^P<0.05 and ^**^P<0.01 using Student's *t*-test for the comparison between vehicle-treated and JWH-015-treated cells, and ^#^*P*<0.01 for the comparison between JWH-015-treated and inhibitor-treated cells.

Ceramide is a sphingolipid messenger that has a relevant role in the regulation of tumour cell fate (Velasco *et al*, 2005; Carpinteiro *et al*, 2008). Recent studies have suggested that apoptosis induced by cannabinoids can be preceded by ceramide accumulation (Gomez del Pulgar *et al*, 2002; Gustafsson *et al*, 2006). To further analyse the apoptotic mechanism of JWH-015, we measured intracellular ceramide concentration in PC-3 cells. As shown in Figure 6A, incubation with JWH-015 for 48 h led to a dose-dependent increase in intracellular ceramide accumulation. This effect was prevented by the CB_2_ antagonist SR2, which indicates that ceramide accumulation was mediated by the activation of CB_2_ (Figure 6B). Ceramide is formed in cellular membranes by *de novo* synthesis through a pathway involving the ceramide synthase or by hydrolysis of sphingomyelin catalysed by acid, neutral and alkaline sphingomyelinases. To gain insight into the origin of JWH-015-induced ceramide increase, cells were incubated in the presence of the ceramide synthase inhibitor, Fumonisin B1, or the sphingomyelinase inhibitor, D609. As shown in Figure 6B, treatment of cells with 10 *μ*M Fumonisin B1, but not with D609, prevented the increase in ceramide concentration, a fact that suggests that ceramide came from *de novo* biosynthesis.

Moreover, treatment with Fumonisin B1 prevented the inhibition of cell growth induced by JWH-015 (Figure 6C), indicating that ceramide biosynthesis was involved in the action of the cannabinoid.

### Signal mechanisms involved in JWH-015-induced prostate PC-3 cell death

**Figure 7:**
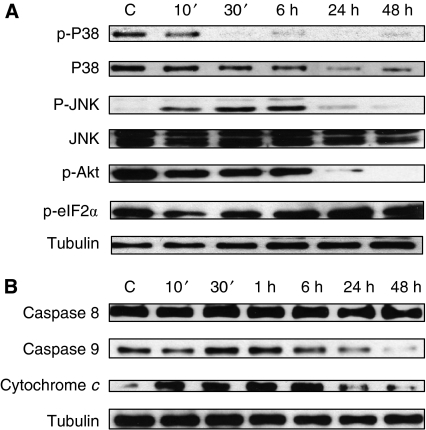
Signalling mechanisms activated by JWH-015 in prostate PC-3 cells. Cells were incubated with 10 *μ*M JWH-015 for different times. (**A**) Phosphorylation levels of p38, JNK, Akt and eIF2*α* were measured by western blot. (**B**) Levels of pro-caspase 8, pro-caspase 9 and cytochrome *c* in the cell cytosol were detected by western blot. Figure shows a representative image of the other three experiments. Tubulin levels are shown as loading control.

To further explore the signalling pathways in which the CB_2_ agonist exerted its effect in prostate PC-3 cells, we studied stress-related MAP kinase cascades activation by western blot. PC-3 cells were treated for different times with 10 *μ*M JWH-015 and then phosphorylated forms of JNK and p-38 kinases, indicative for activated kinases, were detected by western blot. Results in Figure 7A show that JWH-015 activates the stress-signal-related kinase JNK as phosphorylated JNK is increased at 30 min and 1 h of treatment.

Eukaryotic cells respond to stress in their endoplasmic reticulum by phosphorylating the *α*-subunit of translation initiation factor 2 (eIF2*α*). This adaptation inhibits general protein synthesis while promoting translation and expression transcriptional regulators that induce gene expression important for cellular remediation and apoptosis. It has recently been described that cannabinoids promote endoplasmic reticulum stress and autophagy-mediated cell death in glioma cells through the Akt/mammalian target of rapamycin (mTOR) pathway inhibition and eIF2*α* activation (Salazar *et al*, 2009b). To investigate whether JWH-015 regulated this pathway in prostate cells, we treated the cells with the CB_2_ agonist and measured the phosphorylation of Akt in Ser473, which is negatively involved in the autophagy pathway (Todde *et al*, 2009). PC-3 treatment with 10 *μ*M JWH-015 resulted in a long-term AKT phosphorylation decrease that was evident from 6 h of treatment (Figure 7A). This was in concordance with the increase in phosphorylated eIF2*α* (Figure 7A), suggesting that endoplasmic reticulum stress signals were activated by JWH-015 in prostate cells. We further investigated whether JWH-015 induced caspase activation, which can be detected after the decrease in the pro-caspase form and the increase in the proteolytic active form by western blot (Hoffmann *et al*, 2009). Results in Figure 7 show that JWH-015 induced activation of caspase 9, which has been described as an initiator caspase essential in the intrinsic or mitochondrially gated pathway of apoptosis (Johnson and Jarvis, 2004; Denault and Salvesen, 2008). To further confirm the activation of the intrinsic apoptotic pathway, we determined the cytosolic levels of cytochrome *c*, which is released into the cytosol when the cell receives an apoptotic stimulus to trigger programmed death through caspase 9 activation (Ow *et al*, 2008).

### JWH-015 caused regression of prostate cancer xenografts in nude mice

**Figure 8:**
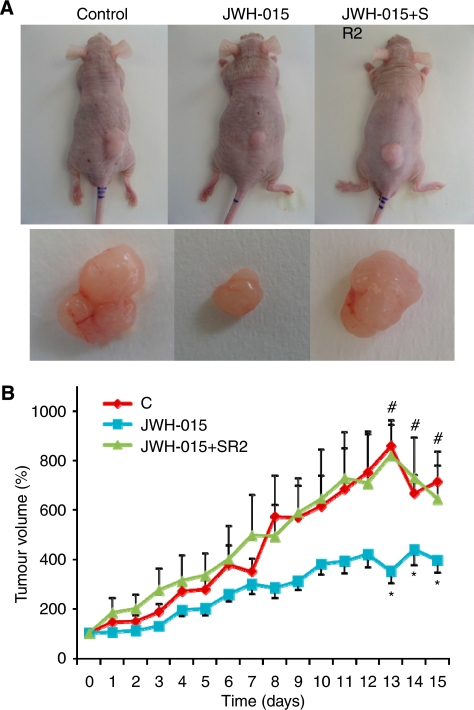
*In vivo* anti-tumoural properties of JWH-015. Athymic nude mice were injected s.c. in the right flank with PC-3 cells and 4 weeks later (day 0) were treated for 15 days with vehicle (control), 1.5 mg kg^−1^ JWH-015 or 1.5 mg kg^−1^ JWH-015 plus 1.5 mg kg^−1^ SR2. Treatments were carried out by injections in the peritumoural area every day. Tumour volumes were measured daily. (**A**) The dorsal side of representative mice and dissected tumours after treatment. (**B**) Tumour growth curve after administration of vehicle (diamonds), JWH-015 (squares) or JWH-015+SR2 (triangles). Results represent the mean±s.e. of eight mice in each group. ^*^P<0.01 *vs* control and ^#^*P*<0.01 *vs* JWH-015, compared by Student's *t*-test.

The above observations of the anti-proliferative effects of JWH-015 were examined in a xenograft model of prostate cancer. Xenograft human prostate tumours were established in nu/nu mice by a s.c. injection. Tumour-bearing animals (∼70 mm^2^) were treated daily with vehicle, 1.5 mg ml^−1^ JWH-015 or 1.5 mg ml^−1^ JWH-015 plus 1.5 mg kg^−1^ SR2. Animals were treated for 15 days and tumour volume was calculated every day. At the end of the experiment, tumours were dissected and weighed. As shown in Figure 8, JWH-015-treated animals had a rapid and dramatic reduction in tumour growth, whereas uncontrolled growth was observed in the control group. The final tumour volume as well as the final tumour weight was significantly lower in the JWH-015-treated group compared with that in the control group (Table 1). Treatment with JWH-015 plus SR2 resulted in a similar growth compared with that in the control group, suggesting that the *in vivo* effect of JWH-015 is also mediated through CB_2_ activation (Figure 8 and Table 1).

## Discussion

Deregulation of cell survival pathways and resistance to apoptosis are widely accepted to be fundamental aspects of tumourigenesis, as evasion of apoptosis may contribute to carcinogenesis, tumour progression and also to treatment resistance. Most current anticancer therapies act by activating cell death pathways in cancer. A number of studies have recently shed light on the role of cannabinoid family members as anticancer agents, allowing the potential development of anti-neoplasic-efficient treatments.

In this study, we demonstrated that the stable anandamide analogue, MET, as well as JWH-015, a cannabinoid receptor type 2-selective agonist, inhibited the proliferation of human prostate cancer PC-3, DU-145 and LNCaP cells. The fact that the anti-proliferative effect of cannabinoids was less pronounced in LNCaP cells is in agreement with previous results showing a higher expression of CB_2_ in PC-3 and DU-145 cells than in LNCaP cells (Sarfaraz *et al*, 2005) and points to a CB_2_-mediated effect. We then demonstrated that CB_2_ was involved in cannabinoid-induced PC-3 cell death, in that blocking its activation with a specific antagonist (SR2) almost totally prevented cell death after incubation of cells with MET and JWH-015. The role of CB_2_ receptors in the anti-proliferative effect of cannabinoids is further supported by results obtained by knocking down CB_2_ mRNA with the use of selective siRNA. There is now a large body of data indicating that the cannabinoid receptor type 2 is linked to various responses related to the proliferation, differentiation and survival of many cell types (Fernandez-Ruiz *et al*, 2007; Svizenska *et al*, 2008). Our observations are consistent with previous reports showing that CB_2_ receptor stimulation is involved in cannabinoid anti-tumour activity *in vitro* and in mice inoculated with tumour xenografts *in vivo* (Caffarel *et al*, 2006; Carracedo *et al*, 2006a; Fernandez-Ruiz *et al*, 2007). As previously proposed, this receptor may function as a signal favouring a non-differentiated, proliferate state of cells (Fernandez-Ruiz *et al*, 2007). In keeping with this notion, increased levels of CB_2_ have been shown in prostate cancer cells compared with normal prostate (Sarfaraz *et al*, 2008), and a correlation between CB_2_ expression and the histological grade of breast tumours has also been observed (Caffarel *et al*, 2006). Activation of the CB_2_ receptor induces apoptosis and reduces tumour growth of glioma (Sanchez *et al*, 2001; Blazquez *et al*, 2008), pancreatic carcinoma (Carracedo *et al*, 2006a) or breast cancer (Caffarel *et al*, 2006). Moreover, recent research shows that the anti-tumour action of cannabinoid receptor agonists in colon cancer cells may be exerted through the CB_2_ receptor more efficiently that through the CB_1_ receptor (Cianchi *et al*, 2008), which is consistent with our results in prostate cells.

Our data show that CB_2_ receptor activation by JWH-015 in prostate PC-3 cells induces *de novo* synthesis of ceramide, which mediates the apoptotic effect of JWH-015 in that the addition of the ceramide synthase inhibitor Fumonisin B1 prevented the induction of apoptosis. Ceramide is a second messenger that has been shown to act as a pro-apoptotic lipid mediator of cannabinoid action (Guzman *et al*, 2001). It has been previously described that, although CB_1_ receptor activation induces acute ceramide increase through sphingomyelin hydrolysis (Sanchez *et al*, 1998), sustained ceramide accumulation through enhanced *de novo* synthesis seems to exert a major effect in CB_2_-induced apoptosis (Gomez del Pulgar *et al*, 2002; Herrera *et al*, 2006; Carracedo *et al*, 2006a; Cianchi *et al*, 2008), which is in good agreement with our data. Many anticancer drugs also induce death by stimulating the generation of ceramide (Claria, 2006; Lin *et al*, 2006). Ceramide mediates the apoptosis of radiation therapy (Moeller *et al*, 2009), and attenuation of ceramide levels confers resistance to radiation in prostate cancer cells (Mahdy *et al*, 2009). Ceramide has been shown to activate a number of enzymes involved in stress signalling cascades, including stress-activated protein kinases such as the Jun kinases JNKs, which are involved in ceramide-mediated apoptosis induction (Ruvolo, 2003). Our data demonstrate that JWH-015 activates JNK in prostate cells, which is in agreement with previous results showing a CB_2_-dependent activation of JNK in microglial cells (Correa *et al*, 2009) and in lung epithelial cells (Sarafian *et al*, 2008). This study also demonstrated that JWH-015 inhibits the Akt–mTOR pathway and activates eIF2*α,* which are involved in autophagy regulation and endoplasmic reticulum stress response, suggesting that these pathways are involved in JWH-015-induced prostate cell death. The release of pro-apoptotic factors such as cytochrome *c* from the mitochondria leads to the formation of a multimeric complex known as apoptosome and initiates caspase activation cascades. The fact that JWH-015 induced cytochrome *c* release into cytosol and activated caspase 9 in prostate PC-3 cells confirms the involvement of apoptosis and points to an activation of the intrinsic apoptotic pathway. An improved understanding of the downstream mechanisms of prostate cell death induced by cannabinoids in prostate cells would offer new opportunities for the development of a combination therapy in the treatment of prostate cancer. Overall, our data show a role for the cannabinoid receptor CB_2_ in the anti-tumour effect of cannabinoids on prostate cells *in vitro* and *in vivo*. There is considerable interest in the application of selective CB_2_ receptor agonists, which are devoid of typical marijuana-like psychoactive properties of CB_1_ agonists, for future cannabinoid-based anticancer therapies. Therefore, our findings point to the potential application of cannabinoid receptor type 2 ligands as anti-tumour agents in prostate cancer.

## Figures and Tables

**Table 1:** Effect of JWH-015 on PC-3 xenograft tumour growth

	**Initial tumour volume (mm^3^)**	**Final tumour volume (mm^3^)**	**% tumour growth**	**Tumour weight (*μ*g)**
Control	77.61±25.82	449.02±72.41	715.08±121.90	221.28±28.08
JWH-015	77.36±13.66	350.09±66.11	396.48±50.10	148.85±29.15
JWH-015+SR2	71.33±14.00	415.75±81.12	644.32±135.45	236.16±47.00
